# Consistent Plant and Microbe Nutrient Limitation Patterns During Natural Vegetation Restoration

**DOI:** 10.3389/fpls.2022.885984

**Published:** 2022-05-19

**Authors:** Yue Xue, Haibin Kang, Yongxing Cui, Sheng Lu, Hang Yang, Jiaqi Zhu, Zhenjie Fu, Chenglong Yan, Dexiang Wang

**Affiliations:** ^1^College of Forestry, Northwest Agriculture & Forestry University, Yangling, China; ^2^Key Laboratory of Forest Cultivation on the Loess Plateau, State Forestry and Grassland Administration, Yangling, China; ^3^College of Urban and Environmental Sciences, Sino-French Institute for Earth System Science, Peking University, Beijing, China

**Keywords:** ecological stoichiometry, nutrient limitation, plant-microbe interaction, plant nutrient, vegetation restoration

## Abstract

Vegetation restoration is assumed to enhance carbon (C) sequestration in terrestrial ecosystems, where plant producers and microbial decomposers play key roles in soil C cycling. However, it is not clear how the nutrient limitation patterns of plants and soil microbes might change during vegetation restoration. We investigated the nutrient limitations of the plant and microbial communities along a natural vegetation restoration chronosequence (1, 8, 16, 31, and 50 years) following farmland abandonment in Qinling Mountains, China, and assessed their relationships with soil factors. The result showed that following natural vegetation restoration, the nitrogen (N) limitation of plant and microbial communities was alleviated significantly, and thereafter, it began to shift to phosphorus (P) limitation at a later stage. Plants showed P limitation 50 years after restoration, while microbial P limitation appeared 31 years later. The changes in plant nutrient limitation were consistent with those in microbial nutrient limitation, but soil microbes were limited by P earlier than plants. Random forest model and partial least squares path modeling revealed that soil nutrient stoichiometry, especially soil C:N ratio, explained more variations in plant and microbial nutrient limitation. Our study demonstrates that the imbalanced soil C:N ratio may determine the soil microbial metabolic limitation and further mediate the variation in plant nutrient limitation during natural vegetation restoration, which provides important insights into the link between metabolic limitation for microbes and nutrient limitation for plants during vegetation restoration to improve our understanding of soil C turnover in temperate forest ecosystems.

## Introduction

Terrestrial forests are considered the main sinks of atmospheric CO_2_, and they play a crucial role in mitigating climate change (Piao et al., 2009; Starke et al., 2021). The restoration of abandoned farmland to natural forests could help to enhance carbon (C) sequestration, which is considered an effective natural measure for improving the ecological environment (Morrien et al., 2017; Deng et al., 2018). In this ecological process, plants fix CO_2_ from the atmosphere through photosynthesis and store a considerable amount of C (Chapin et al., 2011). For soil microorganisms, C sources decomposed in soil organic matter and those provided by plant roots may remain within the system used for new microbial cells growth or lose by microbial respiration (Singh et al., 2004; Sinsabaugh et al., 2013; Manzoni, 2017). Thus, the activities of soil microbial communities combined with those of plants determine C turnover in terrestrial ecosystems (Lal, 2003). During vegetation restoration, the availability of soil resources could restrict the growth of plants and microbial metabolism, as well as influence their interactions, to further affect C sequestration in the soil (Cui et al., 2020; Teixeira et al., 2020). Therefore, it is necessary to understand the variations in nutrient limitations for plants and microbes and their relationship during the vegetation restoration process.

Ecological stoichiometry facilitates the understanding of the flow of energy and nutrients among trophic levels in food webs and is particularly useful for establishing linkages among above- and underground ecosystem components, such as soils, plant tissues, and microbes (Zechmeister-Boltenstern et al., 2015; Yang et al., 2018; Xiao et al., 2021). According to it, many studies have provided insight into the resource limitation of plant growth and microbial processes in diverse ecosystems (Elser et al., 2000; Cleveland and Liptzin, 2007; Heuck et al., 2015; Zechmeister-Boltenstern et al., 2016). In the semiarid region, the soil microbial community was limited by phosphorus (P) throughout the succession, whereas plants were limited by low soil P at the later stage of succession only (Cui et al., 2020). Jiang et al. (2018) and Li et al. (2019) conducted multielement stoichiometry in plant leaves and ecoenzymatic stoichiometry along the Hailuogou Glacier forefield chronosequence and suggested that the limiting factor for plant growth shifted from nitrogen (N) to P with primary succession, whereas the limitation for microbial communities shifted from P to N. Despite all of them, few studies have established the connections between above- and underground nutrient limitations during vegetation restoration. It is well-known that plants and microorganisms depend on each other for the supply of nutrients, where they may engage in nutrient competition and mutualistic interactions (Singh et al., 2004). For example, during the vegetation restoration process, increases in plant residues and rhizosphere exudates provide more readily available carbon to increase the soil microbial abundance, activity, and growth, which may provide more nutrient supplies for plants and soil microbes or aggravate the competition for nutrients between them (Paterson et al., 2010; Bitas et al., 2013; Kuzyakov and Xu, 2013). The biomass compositions of different types of plants vary, so changes in the aboveground community composition alter the proportions of chemical elements in litter fragments that enter the soil and the compositions of soil microbial communities, thereby affecting plant development (Ordoñez et al., 2009). Variations in vegetation types and the densities of plant cover also can strongly affect evapotranspiration and deep percolation and can alter the water-holding ability by influencing the soil structure (Wang et al., 2011; Cui et al., 2020). Previous study reported that variations in soil moisture may affect soil nutrient transportation (Ouyang et al., 2016) and further change the plant growth and microbial activity (Deutsch et al., 2010; Liu et al., 2016). Moreover, the emergence of some plant species with mycorrhiza (e.g., legume species) could alleviate the plant and microbial resource limitations by improving the availability of soil resources (Xiao et al., 2020). Therefore, the relationship between the nutrient supply for aboveground plant growth and the nutrient limitation of underground microorganism activity under the influence of environmental variables during vegetation restoration may be relatively complex. Although previous studies have improved our understanding of plant nutrient stoichiometry dynamics or microbial metabolic limitations during vegetation restoration (Amazonas et al., 2011; Goloran et al., 2015; Zhang et al., 2019), it remains uncertain whether the nutrient limitation of plant communities shows a similar trend with that of soil microbes and whatever mechanisms might be behind any trends that they do show (Zeng et al., 2017; Pang et al., 2020; Zhong et al., 2020; Cui et al., 2021a).

In this study, we selected abandoned land that had undergone vegetation restoration for five different periods (1, 8, 16, 31, and 50 years) in a typical temperate forest ecosystem (Qinling Mountains, China) to investigate the nutrient limitation patterns and their relationships for plants and microbes at the community level, as well as exploring their driving factors during vegetation restoration. Previous study suggested that vegetation restoration may aggravate soil P loss (Lemma et al., 2017). Thus, we hypothesized that the plant and soil microbial metabolism was more likely limited by soil P in the late restoration stages. Moreover, we hypothesized that the plant nutrient limitation varied synergistically with the trend of microbial nutrient limitation during vegetation restoration, which is perhaps due to the interactions between the plants and microorganisms. Finally, we further hypothesized that soil available nutrients could strongly regulate the resource limitation of plant and microbial communities. The main objectives of this study were (1) to explore the variations in plant and soil microbial nutrient limitations following natural vegetation restoration after farmland abandonment; (2) to determine whether the variations in nutrient limitations for plants and microbes were consistent; and (3) to quantify the contributions of different factors to the variations in nutrient limitations for plants and microbial communities.

## Materials and Methods

### Description of the Study Area

This study was conducted at Huoditang Experimental Forest Farm (33°18′–33°28′ N, 108°21′–108°39′ E), Shaanxi Province, China. This forest region is situated in the middle of the Qinling Mountains at altitudes ranging from 1,450 to 2,473 m. This region has a moist temperate climate, with an average annual temperature range of 8°C–10°C (maximum of 28.6°C in July and minimum of –9.5°C in January) and an average annual precipitation range of 1,000–1,200 mm (mostly from July to September). The average frost-free period in this region is 170 days, and the mean total yearly sunshine ranges from 1,100 to 1,300 h. The soil types in the study area are mainly Cambisols, Umbrisols, and Podzols (FAO), and the mean soil depth is 50 cm. In the 20th century, much of the human population of the forest region moved away, and large areas of farmland were gradually abandoned. At present, lands abandoned for different periods of time are widely distributed in the region. The restored vegetation types are mainly grassland, shrub, and natural forest after the secondary succession of the abandoned farmlands. After natural vegetation restoration, the overstory in the study area is dominated by *Quercus aliena var. acuteserrata*, *Pinus tabuliformis*, *P. armandii*, *Q. variabilis*, *Tsuga chinensis*, and *Betula albosinensis*. The main herbs include *Symplocos paniculata*, *Corylus heterophylla*, *Abelia dielsii*, *Lespedeza bicolor*, and *Euonymus alatus*, and the shrubs include *Carex lanceolata*, *Artemisia lavandulaefolia*, *Artemisia gmelinii*, *Thalictrum aquilegifolium* var. *sibiricum*, and *Rubia cordifolia*.

### Experimental Design

**FIGURE 1:**
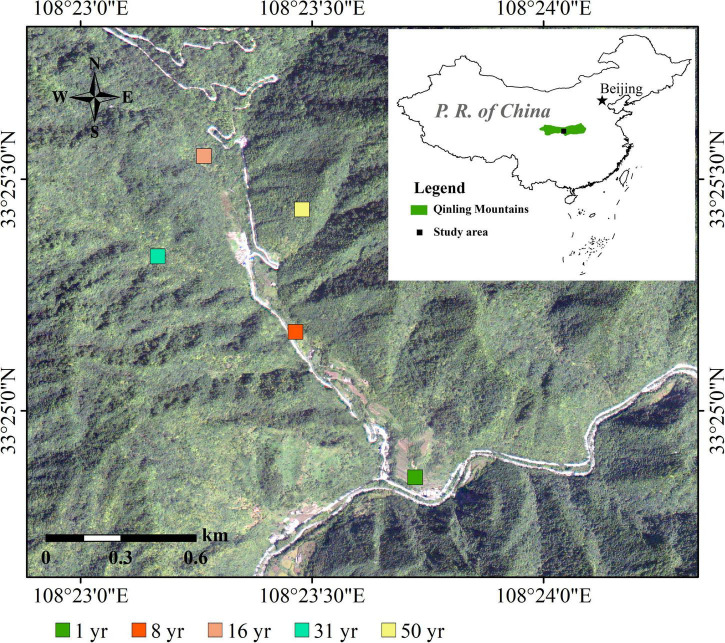
Map of the experimental sites.

In July 2020, five typical abandoned farmlands that had undergone restoration for 1, 8, 16, 31, and 50 years were selected as sample sites in the same watershed (Figure 1 and Table 1). All of the abandoned farmlands were separated by at least 200 m and their elevation, slope aspect, and slope gradient were similar. For each restoration period, five replicate sample plots separated by 30-50 m were selected for subsequent investigation and sampling. In total, 25 sample plots were obtained: five restoration periods × five sample plots. The plots measured 20 m × 20 m, 5 m × 5 m, and 2 m × 2 m for forest, shrub, and herbaceous communities, respectively.

**TABLE 1:** Floristic compositions of the sampling sites.

Restoration stages (yr)	Tree layer		Shrub layer		Herb layer	
	Dominant species	Minor species	Dominant species	Minor species	Dominant species	Minor species
1	/	/	/	/	*Digitaria sanguinalis, Erigeron annuus*	*Artemisia hedinii*
8	/	/	*Rhus chinensis*	*Salix matsudana, Populus simonii*	*Anaphalis aureopunctata*	*Artemisia hedinii, Erigeron annuus*
16	*Toxicodendron Vernicifluum*, *Rhus chinensis*	*Castanea mollissima*	*Rubus flosculosus*	*Berchemia sinica, Desmodium elegans*	*Artemisia argyi*	*Erigeron annuus, Glechoma longituba*
31	*Castanea mollissima*	*Juglans cathayensis, Populus simonii, Rhus chinensis*	*Desmodium elegans, Rubus flosculosus*	*Symplocos paniculata, Corylus heterophylla*	*Carex rigescens, Arthraxon hispidus*	*Artemisia argyi, Pseudocystopteris atkinsonii*
50	*Castanea mollissima, Rhus chinensis*,	*Quercus aliena* var. *acutiserrata, Platycarya strobilacea*	*Dendrobenthamia japonica* var. *chinensis*	*Litsea pungens, Lonicera fragrantissima* subsp. *standishii*	*Carex rigescens, Digitaria sanguinalis*	*Thalictrum aquilegiifolium* var. *sibiricum, Pseudocystopteris atkinsonii*

### Soil and Plant Sampling

We selected replicate points (12 in forest plots and 9 in shrub and grassland plots) along with an “S” shape in each plot for soil sampling. After removing the litter layer, soil samples were collected from a depth of 0–20 cm at each point using a 4.5-cm diameter stainless steel auger. The samples were mixed together to obtain one composite sample per plot. The soil samples were immediately sieved through a mesh measuring < 2 mm, and any roots, litter, animal residues, debris, and stones were removed. Each soil sample was divided into two parts, where one part was naturally air-dried for physicochemical analysis and the other part was stored at 4°C to determine the biological characteristics. Samples for measuring the soil water content and bulk density were obtained from three randomly selected points on the diagonal in each plot at a depth of 0–20 cm using a steel core sampler with a volume of 100 cm^3^. We identified the dominant species along the plant restoration chronosequence and 7–10 plants from each dominant species were selected from each plot. Fully matured and pest-free leaves were collected according to the different levels (high, middle, and low) and different directions (east, south, west, and north) in each plot, and they were then homogenized to obtain one sample.

### Chemical Analyses

The soil water content was determined by oven drying the fresh soil to a constant mass at 105°C. The soil bulk density (BD) was obtained by calculating the ratio of the soil mass relative to the total volume after oven drying to a constant weight at 105°C. The soil pH was measured in a soil:distilled water mixture at a ratio of 1:2.5 (*w*/*v*) using a pH meter (OHAUS ST2100, Shanghai, China). The C contents of soil (soil C) and plant (plant C) samples were determined using the dichromate oxidation method. The N contents of the soil (soil N) and plant (plant N) samples were determined using the Kjeldahl method. Soil total P (soil P), available phosphorus (Olsen-P), and plant phosphorus (plant P) concentrations were measured using the molybdenum antimony reagent colorimetric method. Soil dissolved organic C (DOC) was extracted with 0.5 M K_2_SO_4_, and quantified using a TOC analyzer (TOC–VCPH, Shimadzu, Japan). The soil ammonium (NH_4_^+^-N) and nitrate-nitrogen (NO_3_^–^-N) contents were determined colorimetrically using a continuous flow analyzer. We combined the soil NH_4_^+^-N and NO_3_^–^-N to represent soil available N (TAN) (Bao, 2000). The soil physicochemical properties were shown in Table 2.

**TABLE 2:** Changes in soil physical and chemical properties during vegetation restoration stages.

Restoration stages (yr)	Moisture (%)	pH	BD (g cm^–3^)	soil C (g kg^–1^)	soil N (g kg^–1^)	soil P (g kg^–1^)	soil C:N	soil C:P	soil N:P	DOC (mg kg^–1^)	TAN (mg kg^–1^)	Olsen-P (mg kg^–1^)
1	15.44 ± 1.51c	5.69 ± 0.20b	1.33 ± 0.06a	13.47 ± 2.14b	1.29 ± 0.21b	1.10 ± 0.14b	10.49 ± 0.68c	12.28 ± 1.60b	1.18 ± 0.21bc	41.15 ± 6.44b	7.68 ± 1.82b	12.09 ± 0.74a
8	24.60 ± 1.59b	4.97 ± 0.21c	1.22 ± 0.08b	13.35 ± 1.62b	1.23 ± 0.18b	0.84 ± 0.20c	10.86 ± 0.52b	16.82 ± 4.91b	1.56 ± 0.50b	138.24 ± 29.85a	11.98 ± 3.76b	2.84 ± 0.71cd
16	22.09 ± 1.51b	5.72 ± 0.28b	1.35 ± 0.03a	16.91 ± 4.26b	1.48 ± 0.36b	1.43 ± 0.13a	11.38 ± 0.83b	12.00 ± 3.58b	1.05 ± 0.31c	47.59 ± 6.90b	11.71 ± 3.59b	5.75 ± 1.40b
31	23.29 ± 2.44b	5.52 ± 0.07b	1.37 ± 0.04a	15.72 ± 4.51b	1.38 ± 0.40b	1.11 ± 0.07b	11.40 ± 0.22b	13.99 ± 3.53b	1.23 ± 0.31bc	72.53 ± 13.92b	10.59 ± 1.83b	3.86 ± 1.63c
50	39.73 ± 3.23a	6.11 ± 0.24a	1.20 ± 0.09b	36.80 ± 4.53a	2.80 ± 0.34a	0.52 ± 0.09d	13.14 ± 0.18a	70.77 ± 6.28a	5.39 ± 0.48a	126.89 ± 44.51a	26.92 ± 9.42a	2.03 ± 0.34d

*BD, bulk density; soil C, soil organic carbon; soil N, soil total N; soil P, soil total P; DOC, soil dissolved organic C; TAN, NO_3_^–^-N + NH_4_^+^-N; Olsen-P, soil available P.*

*Different lowercase letters in each column indicate significant differences at P < 0.05 using Duncan tests.*

### Measurements of Microbial Biomass and Extracellular Enzymatic Activity

The soil microbial biomass C, N, and P (MBC, MBN, and MBP, respectively) were determined by chloroform-fumigation extraction (Brookes et al., 1985). The conversion factors used to derive MBC, MBN, and MBP were 0.45, 0.54, and 0.40, respectively (Cui et al., 2020).

The activities of two C-acquiring enzymes [β-1,4-glucosidase (BG) and cellobiohydrolase (CBH)], two N-acquiring enzymes [β-1,4-N-acetylglucosaminidase (NAG) and leucine aminopeptidase (LAP)], and one P-acquiring enzyme [acid phosphatase (AP)] were measured using standard fluorometric techniques (Saiya-Cork et al., 2002; German et al., 2011) according to previously described experimental procedures (Cui et al., 2021a). Briefly, the five enzyme activities were measured fluorometrically using a 200 μM solution of substrates labeled with 4-methylumbelliferone (MUB) or 7-amino-4-methylcoumarin (AMC). In total, 50 μl of 50 mM buffer were pipetted into wells of black 96-well microplates to serve as blanks (buffer + slurry), and 200 μl of 50 mM buffer were pipetted into wells as the reference standard (buffer + standard) and negative control (buffer + substrate) (eight analytical replicates per soil per assay). A total of 1 g of fresh soil was homogenized in 125 ml of 50 mM buffer on a constant temperature (25°C) shaker for 2 h. The soil suspension (slurry) was continuously stirred as 200 μl aliquots were dispensed into the microplate wells that served as the sample assay (16 analytical replicate suspensions for each sample per assay) and as the blank and quench standard (slurry + standard) (eight analytical replicates each). In total, 25 μl of a fluorescence standard solution (10 μM 4-methylumbelliferone-MUB or 7-amino-4-methylcoumarin-AMC for the LAP assay) were dispensed into microplate wells that served as a reference standard (buffer + standard) and as a quench standard. Finally, the sample assays (slurry + substrate) and negative controls (buffer + substrate) also received 25μl of a 200 μM substrate solution in a final reaction volume of 125 μl. Prepared plates were incubated at 25°C in the dark for up to 4 h following the substrate addition. After incubation, 10 μl of 0.5 M NaOH was added to each well to terminate the reactions, and fluorescence was measured using a microplate reader (Tecan Infinite M200pro, Switzerland) with 365 nm excitation and 450 nm emission filters. The measurements of fluorescence for the negative controls, blanks, and quench standards were corrected, and the enzymatic activity was expressed as nmol g^–1^ h^–1^.

### Quantification of Microbial Nutrient Limitation

The classic threshold element ratio (TER) model defines the elemental C:N or C:P ratio at which control of microbial metabolism switches from energy (C) to nutrient (N/P) availability, but cannot distinguish the single most limiting nutrient (Sterner and Elser, 2002). A new model (TER_*L*_) based on the TER principle was developed to redefine the boundary between P and N limitations. TER_*L*_ model defines the microbial N or P limitation by scaling the TER. We calculated a new TER model (TER_*L*_) to identify which nutrient has the strongest limitation with the following equations (Cui et al., 2021b):


$${\text{TER}}_{\text{L}}=\left(\Delta \text{TER}2_{\text{C}:\text{P}}/{\text{B}}_{\text{C}:\text{P}}\right)-\left(\Delta \text{TER}2_{\text{C}:\text{N}}/{\text{B}}_{\text{C}:\text{N}}\right)$$

$$\Delta \text{TER}2_{\text{C}:\text{X}}=L_{\text{C}:\text{X}}-{\mathrm{TER}}_{\text{C}:\text{X}}$$

$${\text{TER}}_{\text{C}:\text{X}}=\left({\text{EEA}}_{\text{C}:\text{X}}\times B_{\text{C}:\text{X}}\right)/\text{z}0$$

where X represents N or P; TER_*C:X*_ is the TER calculated based on the C/N or C/P ratio; L_*C:X*_ is the C/N or C/P ratio of the availability of soil resources; EEA_*C:X*_ represents the between C- and N (or P)-acquiring enzyme activities [i.e., (BG + CBH)/(NAG + LAP) or (BG + CBH)/AP]; B_*C:X*_ is the C/N or C/P ratio of the microbial biomass; z0 = e intercept in the standard major axis (SMA) for ln (BG + CBH) vs. ln (NAG + LAP) or ln AP (Supplementary Table 1). TER_*L*_ > 0 represent microbial P limitations, whereas < 0 represent N limitations.

### Statistical Analyses

One-way analysis of variance (ANOVA) was conducted to determine the effects of restoration time on the soil physicochemical properties, plant elements and their stoichiometry, and TER_*L*_ values during vegetation restoration. Significantly different means were then compared using Tukey’s multiple comparisons test (*P* < 0.05) in R. The normalization constant z_0_ was obtained from the standardized major axis (Type II) regressions (Supplementary Table 1). Pearson’s correlation coefficients were calculated to examine the relationships among the soil physicochemical properties, plant elements and their stoichiometry, microbial biomasses and their stoichiometry, and TER_*L*_ values. Principal component analysis was used to determine the overall differences in soil properties, TER_*L*_ and plant N:P during vegetation restoration and the relationships between soil properties, TER_*L*_ and plant N:P. A random forest test could quantify the correlation importance of variables in each input model, which is performed to identify the main factors (Zeng et al., 2021). To tease apart the relative importance of various soil variables on TER_*L*_ and plant N:P ratio, we used the increased node purity (IncNodePurity) of the variables. Partial least squares path modeling (PLS-PM) was conducted to further identify possible pathways for variables that controlled plant and microbial nutrient limitations. All statistical analyses were performed using R software (version 3.6.2).

## Results

### Ecological Stoichiometry in Plants During Restoration

**FIGURE 2:**
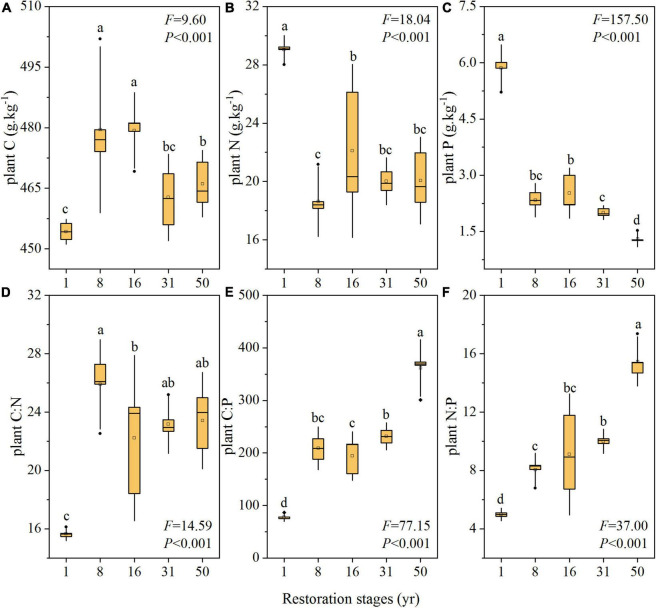
Changes in plant nutrient contents and ratios during vegetation restoration stages. plant C, plant C concentration; plant N, plant N concentration; plant P, plant P concentration. Different lowercase letters indicate significant differences at *P* < 0.05 using the Duncan tests.

Vegetation restoration led to significant variations in the plant nutrient concentrations (*P* < 0.001, Figures 2A–C). The plant C concentration increased significantly initially and then decreased over the restoration gradient, with the highest value after 8 years (479.49 ± 13.70 g kg^–1^, Figure 2A). By contrast, the N concentration decreased significantly initially and then increased with the restoration time, where the highest value was obtained after 1 year and the lowest after 8 years (29.04 ± 0.65 and 18.63 ± 1.60 g kg^–1^, respectively, Figure 2B). The P concentration decreased significantly over time, where the minimum value (1.30 ± 0.14 g kg^–1^) occurred after 50 years (Figure 2C). In addition, the plant element ratios changed significantly with the restoration time (*P* < 0.001, Figures 2D–F). The C:N ratio increased significantly initially and peaked after 8 years (25.90 ± 2.04), then decreased gradually (Figure 2D). Both C:P and N:P increased significantly with the restoration time, where they ranged from 77.93 to 361.74 and from 4.98 to 15.47, respectively (Figures 2E,F).

### Soil Microbial Metabolic Limitations During Restoration

**FIGURE 3:**
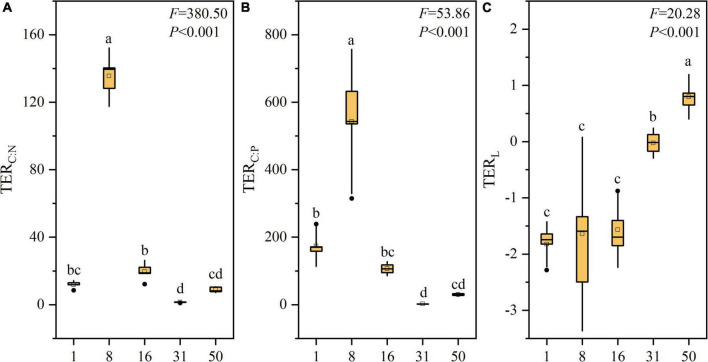
Changes in TER_*L*_ during vegetation restoration stages. Different lowercase letters indicate significant differences at *P* < 0.05 using the Duncan tests.

Vegetation restoration significantly affected TER_*C:N*_ and TER_*C:P*_ values (*P* < 0.001, Figures 3A,B). TER_*C:N*_ increased significantly initially and then decreased over the restoration gradient, with the highest value after 8 years. TER_*C:P*_ also increased significantly initially and peaked after 8 years, before then decreasing gradually. In addition, the TER_*L*_ changed significantly with the restoration time (*P* < 0.001, Figure 3C). TER_*L*_ increased significantly over time, where the TER_*L*_ value in 1, 8, 16, 31 and 50 years were –1.83 ± 0.27, -1.64 ± 1.15, -1.57 ± 0.45, -0.03 ± 0.18, and 0.80 ± 0.26, respectively. The results indicated that soil microbial N limitation decreased significantly in the first 31 years of farmland abandonment and thereafter shifted to microbial P limitation.

### Relationships Between Soil Properties, Microbial Metabolic Limitation, and Plant Nutrient Stoichiometry

**FIGURE 4:**
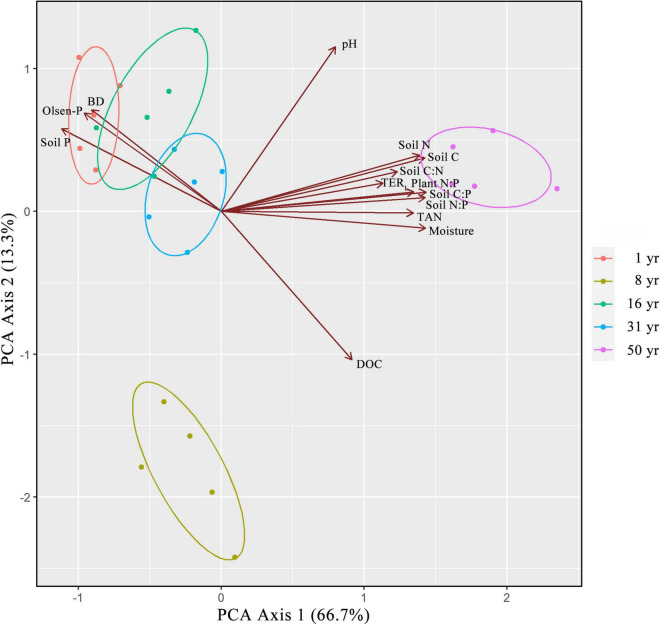
Variable ordination diagram of PCA for the first two principal component axes. plant N:P, the ratio of plant N and plant P concentration; soil C, soil organic carbon; soil N, soil total N; soil P, soil total P; DOC, soil dissolved organic C; TAN, NO3^–^-N + NH4^+^-N; Olsen-P, soil available P; Moisture, soil moisture; BD, bulk density.

The results of PCA showed that soil properties, TER_*L*_, and plant N:P explained 80.0% of the vegetation restoration variations through two main corrected variable groups (PC1 = 66.7%; PC2 = 13.3%). The overall differences in soil properties, TERL, and plant N:P significantly changed during vegetation restoration. PC1 strongly distinguished the 50-year site from other sites. The discrimination of 8-year site from other sites was strongly influenced by PC2. The results of PCA also showed that TER_*L*_ had highly association with plant N:P. Moreover, TER_*L*_ and plant N:P had similar corrections with the soil factors (Figure 4).

**FIGURE 5:**
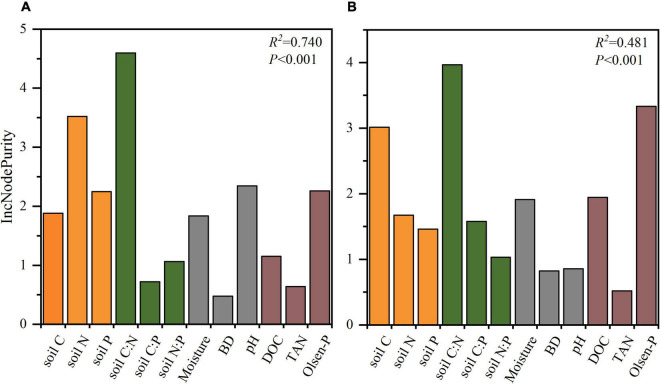
The effects of soil physical and chemical properties on plant N:P **(A)** and TER_*L*_ **(B)** during vegetation restoration stages using random forest test. soil C, soil organic carbon; soil N, soil total N; soil P, soil total P; DOC, soil dissolved organic C; TAN, NO_3_^–^-N + NH_4_^+^-N; Olsen-P, soil available P; Moisture, soil moisture; BD, bulk density.

The results from the random forest test were used to identify the effects of factors on the plant N:P ratio and TER_*L*_. The results showed that soil C:N ratio was the most important factor in plant N:P ratio, followed by soil N, pH, Olsen-P, soil P, soil C, Moisture, DOC, soil N:P ratio, soil C:P ratio, TAN, and BD (Figure 5A). The random forest test also showed that soil C:N ratio was the main factor in TER_*L*_, followed by Olsen-P, soil C, DOC, Moisture, soil N, soil C:P ratio, soil P, soil N:P ratio, pH, BD, and TAN (Figure 5B).

**FIGURE 6:**
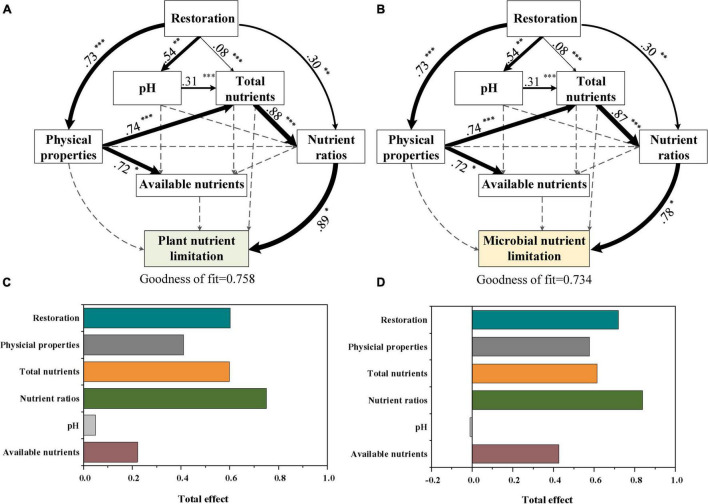
Cascading relationships between plant nutrient limitation and microbial nutrient limitation with restoration time and soil physicochemical properties. Partial least squares path modeling disentangling major pathways of the influences of restoration times, soil pH, physical properties, total nutrients, nutrient ratios, and available nutrients on plant N:P **(A,C)** and TER_*L*_ **(B,D)** during vegetation restoration stages. Black and red arrows indicate positive and negative flows of causality (*P* < 0.05), respectively. Numbers on the arrow indicate significant standardized path coefficients. *R*^2^ indicates the variance of dependent variable explained by the model. Physical properties include soil moisture and bulk density. Total nutrients include soil C, soil organic carbon; soil N, soil total N; and soil P, soil total P. Nutrient ratios include the soil C:N ratio, soil C:P ratio, and soil N:P ratio. Available nutrients conclude DOC, soil dissolved organic C; TAN, NO_3_^–^-N + NH_4_^+^-N; and Olsen-P, soil available P.

Moreover, the PLS-PM identified direct and indirect effects of restoration time, soil physical properties, pH, total nutrients, and their ratios as well as available nutrients on plant N:P ratio and TER_*L*_ (Figures 6A,B). The restoration time (0.72), physical properties (0.58), total nutrient contents (0.62), nutrient ratios (0.84), and available nutrient contents (0.43) had positive total effects on the plant N:P ratio, while the pH of –0.01 showed negative total effects on it (Figure 6C). However, all of the restoration time (0.60), physical properties (0.41), pH (0.05), total nutrient contents (0.60), nutrient ratios (0.75), and available nutrient contents (0.22) had positive effects on TER_*L*_ (Figure 6D).

## Discussion

### Trends of Plant and Microbial Nutrient Limitation During Vegetation Restoration

Microbial communities may be subject to diverse restrictions on soil nutrients under different environmental conditions (Cui et al., 2021a). TER_*L*_ can be used to identify which element has the strongest limitation for microbial community growth (Cui et al., 2021b). In this study, microbial metabolism was N-limited at the early stage of vegetation restoration (< 31 years) and the microbial N limitation decreased significantly, thereafter shifted to microbial P limitation (Figure 3C). This finding was consistent with the result obtained in the study by Jiang et al. (2019), reporting that soil microorganisms were limited by N at the early successional stage, while P was the main limitation factor at a later stage in the Hailuogou Glacier Chronosequence. Zhong et al. (2020) comparing sites along the *Robinia pseudoacacia* afforestation chronosequence on the Loess Plateau of China, reported that microbial communities were co-limited by N and P, and they then became more limited by P. However, Wu et al. (2021) demonstrated that microbial metabolic P limitation was strong initially, but it then decreased in microbial communities during secondary plant succession on the Loess Plateau, China. Vegetation restoration results in variations in soil substrate, plant composition, and environmental parameters that could strongly influence the microbial metabolic limitation (Bitas et al., 2013; Xu et al., 2017; Moreau et al., 2019). In this study, the soil TAN content and soil N:P ratio increased significantly, while the concentration of Olsen-P decreased with vegetation restoration (Table 2). The lower content of soil TAN and soil N:P ratio in the early stage of vegetation restoration could result in the N limitation of microbial communities. As for the vegetation restoration, the improvement of available N derived from the input of plant residues with the higher N:P ratio and the emergence of legume species alleviated the microbial N limitation (Tables 1, 2). However, the decrease in P release and soil erosion could intensify the loss of soil P, and thus contribute to the soil P limitation at the late stage of vegetation restoration (Lemma et al., 2017).

Moreover, we found that the plant N:P ratio varied from 4.98 to 10.00 in the first 31 years of vegetation restoration, which might indicate that the growth of the plant was limited by N at the early stage (Figure 2F) (Koerselman and Meuleman, 1996). This result was confirmed by the studies of Wang et al. (2018) and Pang et al. (2021), reporting that there was limited N availability in temperate forests. We also found that the plant N:P ratio increased significantly over time with the highest value close to 16 obtained after 50 years (Figure 2F). These results suggested that the N limitation of the plant was alleviated gradually and thereafter shifted to P limitation, which was also reflected in the increased plant N concentration and decreased plant P (Figures 2B,C). Similarly, Wang and Zheng (2021) demonstrated the N limitation of plant growth at the early stage of vegetation succession and thereafter a P limitation occurrence. One of the reasons might be that the dilution effect of soil C and N accumulation on soil P could cause or intensify the P limitation in the later stage of vegetation restoration (Groenigen et al., 2006). Moreover, the distinct beta diversities of the *apr-* and *phoD-*harboring bacteria could cause a steady increase in soil available N and a general decrease in available P, which may further affect the plant nutrient limitation as ecosystem restoration proceeds (Wang et al., 2020; Xu et al., 2020). In brief, our results suggest that the nitrogen limitation of plant and microbial communities was alleviated significantly and thereafter began to shift to P limitation at the later stages of vegetation restoration, supporting our first hypothesis, which is that plant and soil microbial metabolism are more likely limited by soil P in the late restoration stages.

In particular, we observed that TER_*L*_ had a positive correlation with the plant N:P ratio according to principal component analysis and linear regression analysis (Figure 4, Supplementary Figures 1, 2). These findings suggest that there was coordinated variation between microbial metabolic limitation and plant nutrient limitation at the community level during vegetation restoration, which seems to support our second hypothesis. Plant nutrient supply is mainly relied on the microbial decomposition and mineralization of soil organic matter by secreting exoenzymes (Kastovska et al., 2015). Thus, the nutrient status of plants and microbes may be coupled in time. We also found that compared with soil microorganisms, plants were limited by N for longer (Figures 2F, 3C). One of the reasons might be that during vegetation restoration, the increases in plant residues and rhizosphere exudates result in more readily available carbon to increase the abundance, activity, and growth of microorganisms, which could aggravate the competition for nutrients between microorganisms and plants (Paterson et al., 2010; Kuzyakov and Xu, 2013). In this study, analysis based on Pearson’s correlation coefficients also showed that the N:P ratio in plants had a negative correlation with that of the microbial community (Supplementary Figure 2). Due to more rapid growth rates and higher surface-area-to-volume ratios than plant root hairs, microorganisms could outcompete the roots for inorganic N (Rosswall, 1982). For example, Liu et al. (2016) found that the amount of N taken up by microbes was at least seven times that of plants in a temperate grassland. Moreover, the relative distributions of roots and microorganisms in the soil, mineralization pathways for elements, plant-plant interactions, and spatiotemporal variations could regulate the competition between plants and microorganisms for nutrients (Zak et al., 1990; Kaye and Hart, 1997; Manzoni and Porporato, 2007; Song et al., 2007; Xu et al., 2011). Thus, microbial-plant interactions and uncertainties regarding plant and microbial nutrient competition may lead to a slight mismatch in the synergy of nutrient limitations for plants and microbes.

### Drivers of Plant and Microbial Nutrient Limitation During Vegetation Restoration

The soil quality improved and the organic matter inputs increased during vegetation restoration, which further affected the acquisition of nutrients by plants and microbes. In this study, we found that the nutrient limitation of plants and soil microorganisms was strongly affected by soil total nutrient stoichiometry, especially the soil C:N ratio (Figures 4, 5, 6C,D). These results seem inconsistent with our third hypothesis, which is that soil available nutrients might be the main factor affecting the resource limitation of plant and microbial communities. One of the potential reasons is that there is a certain C:N threshold ratio that soil must reach to be conducive for the growth of microorganisms (Zhong et al., 2020). The unbalance of soil elemental stoichiometry might cause the variation of plant nutrient stoichiometry and microbial metabolic limitation (Deng et al., 2019; Qiu et al., 2020; Cui et al., 2021a; Xiao et al., 2021). For example, Zhang et al. (2018) evaluated the factors that influence leaf P stoichiometry along a chronosequence of *Metasequoia glyptostroboides* forests and found that the plant N:P ratio was impacted more by soil stoichiometry. Cui et al. (2019) reported that soil C:N, C:P, and N:P ratios had a strong influence on microbial nutrient acquisition in grasslands, which might be due to the efficient influence of soil C:N:P stoichiometry on the structure and activity of microbial communities. Moreover, Zhang et al. (2022) found that the soil substrate C:N ratio changed more consistently with the trend of soil microbial N limitations than other stoichiometric ratios. We also found that the soil substrate (soil C, soil N, soil P, TAN, and Olsen-P) and soil physical properties (moisture and pH) had strong associations with TER_*L*_ and plant N:P ratio (Figure 4 and Supplementary Figure 2). Thus, the soil total nutrients, soil available nutrients, and soil physical properties significantly affected plant and microbial nutrient limitation, as also found in previous studies (Price and Sowers, 2004; Bowles et al., 2014; Zhan et al., 2017; Deng et al., 2019; Liu et al., 2020; Xiao et al., 2020; Li et al., 2021). Furthermore, PLS-PM showed that the restoration time, soil physical properties, pH, and total nutrients directly determined the soil nutrients ratios and caused the nutrient limitation of plant and microbial communities (Figures 6A,B). Consequently, we suggest that the soil nutrient stoichiometry, especially the soil C:N ratio, directly determined nutrient limitation for plant and microbial communities, and it had the greatest effect during vegetation restoration.

## Conclusion

Vegetation restoration may increase soil N availability whereas decrease the P in the temperate forest. During the vegetation restoration, the N limitation of plant communities alleviated significantly and thereafter began to shift to P limitation at the later stage (50 years). Meanwhile, the N limitation of soil microorganisms also alleviated significantly and thereafter began to shift to P limitation after 31 years. Moreover, plant N:P ratio had a significantly positive correlation with TER_*L*_. These results indicated that the changes in plant nutrient limitation were consistent with those in microbial nutrient limitation, but soil microbes were limited by P earlier than plants. Soil nutrient stoichiometry, especially soil C:N ratio, was the key factor that affected nutrient limitation for plant and microbial communities. This finding confirmed that soil C:N may determine the microbial nutrient acquirement and further mediate the variation in plant nutrient stoichiometry. Our findings provide important insights into the links between microbial metabolic limitation and plant nutrient limitation during vegetation restoration to improve our understanding of soil C turnover in temperate forest ecosystems.

## Data Availability Statement

The original contributions presented in the study are included in the article/Supplementary Material, further inquiries can be directed to the corresponding author.

## Author Contributions

DW conceptualized the study and contributed to funding acquisition, resources, and supervision. YX, HK, SL, HY, JZ, ZF, and CY contributed to formal analysis and investigation. YX and HK contributed to methodology and wrote the original draft. DW and YC contributed to writing, reviewing, and editing the manuscript. All authors contributed to the article and approved the submitted version.

## Conflict of Interest

The authors declare that the research was conducted in the absence of any commercial or financial relationships that could be construed as a potential conflict of interest.

## Publisher’s Note

All claims expressed in this article are solely those of the authors and do not necessarily represent those of their affiliated organizations, or those of the publisher, the editors and the reviewers. Any product that may be evaluated in this article, or claim that may be made by its manufacturer, is not guaranteed or endorsed by the publisher.
