# Supplementary material for: Consistent Plant and Microbe Nutrient Limitation Patterns During Natural Vegetation Restoration
Source: Front Plant Sci. 2022 May 19;13:885984. doi: 10.3389/fpls.2022.885984 (PMC9161215; doi:10.3389/fpls.2022.885984)
Supplement: Supplementary file 1 [file Data_Sheet_1.docx]

Supplementary Material

## Supplementary Table

**Table S1** The intercepts and slopes of C-acquiring versus N (or P)-acquiring enzyme activity relationships for five restoration stages using standardized major axis (Type II) regression.

| Regression | Restoration stages (yr) | Intercept | 97.5%CI | | Slope | 97.5%CI | | *R^2^* | *P* |
| --- | --- | --- | --- | --- | --- | --- | --- | --- | --- |
|  |  |  | lower | upper |  | lower | upper |  |  |
| ln(BG+CBH) *vs*. ln(NAG+LAP) | 1 | 1.52 | -0.73 | 2.64 | 0.96 | 0.47 | 1.94 | 0.83 | 0.03 |
|  | 8 | -0.88 | -6.64 | 1.63 | 1.75 | 0.76 | 4.01 | 0.74 | 0.06 |
|  | 16 | 1.00 | -2.70 | 2.76 | 1.23 | 0.53 | 2.37 | 0.80 | 0.04 |
|  | 31 | 3.25 | 1.31 | 3.88 | 0.29 | 0.09 | 0.91 | 0.43 | 0.23 |
|  | 50 | 1.11 | -1.44 | 2.53 | 0.82 | 0.46 | 1.48 | 0.89 | 0.02 |
| ln(BG+CBH) *vs*.  lnAP | 1 | -1.07 | -5.94 | 1.34 | 1.22 | 0.60 | 2.45 | 0.83 | 0.03 |
|  | 8 | -2.20 | -13.11 | 1.57 | 1.52 | 0.53 | 4.39 | 0.52 | 0.17 |
|  | 16 | -0.99 | -7.48 | 1.94 | 1.26 | 0.57 | 2.79 | 0.77 | 0.05 |
|  | 31 | 3.13 | 1.48 | 3.77 | 0.25 | 0.10 | 0.63 | 0.65 | 0.10 |
|  | 50 | 0.40 | -3.72 | 2.42 | 0.86 | 0.42 | 1.75 | 0.82 | 0.03 |

## Supplementary Figures





**Fig. S1** Correlations between plant N:P ratio and TER_L_. Note: full line indicates the model fits between plant N:P ratio and TER_L_. Grey areas are the 95% confidence intervals of the model.


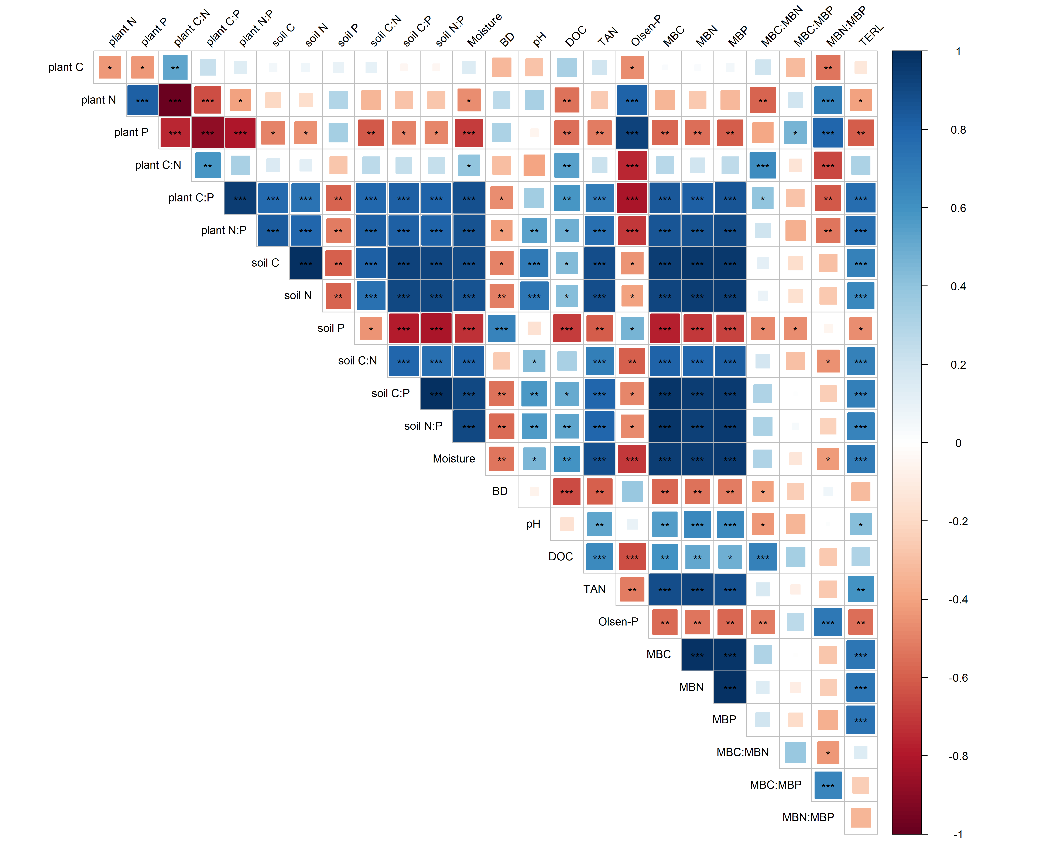


**Fig. S2** Heat map based on the correlation among soil physicochemical properties, plant elements and their stoichiometry, microbial biomasses and their stoichiometry and TER_L_. **Note:** plant C, plant C concentration; plant N, plant N concentration; plant P, plant P concentration; soil C, soil organic; soil N, soil total N; soil P, soil total P; DOC, soil dissolved organic C; TAN, NO3^-^-N + NH4^+^-N; Olsen-P, soil available P; Moisture, soil moisture; BD, bulk density; MBC, soil microbial biomass C; MBN, soil microbial biomass N; MBP, soil microbial biomass P; TRE_L_, soil microbial nutrient limitation. * Correlation is signiﬁcant at *P* < 0.05 (two-tailed); ** Correlation is signiﬁcant at *P* < 0.01 (two-tailed); *** Correlation is signiﬁcant at *P* < 0.001 (two-tailed).
